# Alternative scenarios: harnessing mid-level providers and evidence-based practice in primary dental care in England through operational research

**DOI:** 10.1186/s12960-015-0072-9

**Published:** 2015-09-15

**Authors:** Kristina L. Wanyonyi, David R. Radford, Paul R. Harper, Jennifer E. Gallagher

**Affiliations:** King’s College London Dental Institute, Division of Population and Patient Health, Bessemer Road, London, UK; King’s College London Dental Institute, Teaching Division, Guys Tower, Guys Hospital, London, UK; University of Portsmouth Dental Academy, Hampshire Terrace, Portsmouth, UK; Cardiff University, School of Mathematics, Cardiff, UK

## Abstract

**Background:**

In primary care dentistry, strategies to reconfigure the traditional boundaries of various dental professional groups by task sharing and role substitution have been encouraged in order to meet changing oral health needs.

**Aim:**

The aim of this research was to investigate the potential for skill mix use in primary dental care in England based on the undergraduate training experience in a primary care team training centre for dentists and mid-level dental providers.

**Methods:**

An operational research model and four alternative scenarios to test the potential for skill mix use in primary care in England were developed, informed by the model of care at a primary dental care training centre in the south of England, professional policy including scope of practice and contemporary evidence-based preventative practice. The model was developed in Excel and drew on published national timings and salary costs. The scenarios included the following: “No Skill Mix”, “Minimal Direct Access”, “More Prevention” and “Maximum Delegation”. The scenario outputs comprised clinical time, workforce numbers and salary costs required for state-funded primary dental care in England.

**Results:**

The operational research model suggested that 73% of clinical time in England’s state-funded primary dental care in 2011/12 was spent on tasks that may be delegated to dental care professionals (DCPs), and 45- to 54-year-old patients received the most clinical time overall. Using estimated National Health Service (NHS) clinical working patterns, the model suggested alternative NHS workforce numbers and salary costs to meet the dental demand based on each developed scenario. For scenario 1:“No Skill Mix”, the dentist-only scenario, 81% of the dentists currently registered in England would be required to participate. In scenario 2: “Minimal Direct Access”, where 70% of examinations were delegated and the primary care training centre delegation patterns for other treatments were practised, 40% of registered dentists and eight times the number of dental therapists currently registered would be required; this would save 38% of current salary costs cf. “No Skill Mix”. Scenario 3: “More Prevention”, that is, the current model with no direct access and increasing fluoride varnish from 13.1% to 50% and maintaining the same model of delegation as scenario 2 for other care, would require 57% of registered dentists and 4.7 times the number of dental therapists. It would achieve a 1% salary cost saving cf. “No Skill Mix”. Scenario 4 “Maximum Delegation” where all care within dental therapists’ jurisdiction is delegated at 100%, together with 50% of restorations and radiographs, suggested that only 30% of registered dentists would be required and 10 times the number of dental therapists registered; this scenario would achieve a 52% salary cost saving cf. “No Skill Mix”.

**Conclusion:**

Alternative scenarios based on wider expressed treatment need in national primary dental care in England, changing regulations on the scope of practice and increased evidence-based preventive practice suggest that the majority of care in primary dental practice may be delegated to dental therapists, and there is potential time and salary cost saving if the majority of diagnostic tasks and prevention are delegated. However, this would require an increase in trained DCPs, including role enhancement, as part of rebalancing the dental workforce.

## Background

In England, planners of state-funded care within the National Health Service (NHS) actively support the development of skill mix through maximizing on skills of dental auxiliaries or dental care professionals (DCPs) and larger dental teams, in the interests of patient care [1]. In addition, the dental team has been expanded and is increasingly professionalized compared with other countries. DCPs, as they are referred to in the UK, include dental hygienists, dental nurses, orthodontic therapists, dental hygiene therapists, clinical dental technicians and dental technicians [2]. Of particular focus has been the expansion of dental hygienist and therapist roles to include direct access and diagnosis in order to improve state-funded primary dental care [1]. The term DCP is peculiar to the UK; therefore, the term mid-level provider has been used to describe the cadre of health worker who would be involved in the model of practice investigated in this study.

Mid-level provider is a universal term and has been described by the WHO as “a group of cadres who are trained for 2–5 years to acquire basic skills in diagnosing, managing common conditions, and preventing disease” [3]. Dovlo [4] describes them as “health cadres who have been trained for shorter periods and required lower entry educational qualifications, to whom are delegated functions and tasks normally performed by more established health professionals with higher qualifications”. They have also been considered as effective contributors to routine care and, if deployed appropriately, can contribute to a more efficient human resource skill mix [3]. Based on these descriptions and the context of this work which investigated the potential for delegation of human resource scenarios in routine primary dental care, the term mid-level dental providers was viewed as an appropriate description of the role and scope of practices of the providers targeted in this research.

Emphasis is also being placed on the role of primary care dental practitioners in delivering evidence-based prevention, supported by the whole dental team [5-7]. This is in recognition of the changing oral health needs within the population in England [8,9]. Therefore, changes to professional regulations by the General Dental Council have included “Direct Access” [2,10], whereby patients are now able to access recently trained dental hygienists and dental therapists without first seeing a dentist.

Of particular focus in the expanding dental team are the dental therapists who have the most overlap in scope of practice with dentists as they are able to undertake a range of restorative and preventive tasks on both adults and children. Others in the group are dental hygienists who help patients maintain their oral health by preventing and treating periodontal disease and promoting good oral health practice and dental nurses. The latter are now able to gain additional skills in oral health education and the application of fluoride varnish [2]. Research evidence suggests that there are no significant issues of patient safety resulting from direct access to DCPs [11], but strong evidence of improved access to dental care, cost benefits to patients and increased patient satisfaction. Other studies suggest that dental therapists are efficient in screening for disease [12]. It is, therefore, other factors, which are associated with cost and the organization of the expanded dental team within the current primary dental care system in England, that are of concern in advancing dental skill mix [13,14].

Historically, evidence of skill mix usage in primary dental care in England has been limited, and there has been a call for more research to aid in better understanding of how skill mix is used in general dental practice [15,16]. The limited research in the field has focussed on the challenges of cost and how to attain technically efficient models of dental skill mix [14,16]. Other research has demonstrated positive findings in regard to acceptability and quality of care from DCPs, with studies suggesting that the public are comfortable receiving care from DCPs [17-20]. In respect to the technical competence, no notable differences between dentists and dental auxiliaries have been identified; however, a recent Cochrane review [21] suggests there is a paucity of high-quality evaluations of the relative effectiveness, cost-effectiveness and safety of dental auxiliaries compared with dentists in performing clinical tasks and further reiterates the need for more research in the subject.

In light of the changing oral health needs and move towards maintaining oral health, it can be suggested that informative research that investigates the use of skill mix implementation is necessary. Research methods such as scenario testing are known to be beneficial in aiding an understanding of alternative events in order to inform future planning [22]. This type of research could advance the knowledge on the real potential for dental skill mix with respect to changing oral health needs [23]. In the past, researchers have modelled a variety of future scenarios using operational research (OR) techniques that incorporate the use of mid-level dental providers and estimated workforce capacity for different patient groups within changing demography [23-25]. With scenario testing, a range of evidence-based policy alternatives can be tested and the learning can be used to plan appropriately for workforce numbers. Therefore, the objective of this study was to examine the implications of alternative models of dental skill mix and evidence-based preventive practice in the delivery of NHS primary dental care in England using OR techniques testing alternative scenarios.

## Methods

An operational supply and demand modelling exercise was undertaken, which simulated alternative scenarios of skill mix use in state-funded primary dental in England. “OR helps to identify solutions to problems that limit quality, efficiency and effectiveness, or to determine which alternative service delivery strategy would yield the best outcome” [26]. In this instance, OR was used to test scenarios related to implementation of regulation changes that expand the scope of practice of mid-level dental providers and improved delivery of evidence-based prevention. The supply and demand model components were informed by findings from a previous study of a primary care educational facility which operates a regular live state-funded primary care contract in the South of England. The facility promotes the training of dental and dental care professional students in an integrated manner, sharing clinical tasks. It is also unencumbered by payment issues, as care is provided free at the point of delivery and there is a philosophy of using the skill mix of the dental team. The findings on the access patterns of patients to the facility and the sharing of tasks between dental students and mid-level dental provider students have already been published [27,28]; they identified patterns of access expected in primary care and quantify the sharing of tasks between the two student dental provider groups. The OR model also included validated treatment timing data from the British Dental Association (BDA) inquiry in 1999 [29]. For three treatments, a panel inquiry was undertaken with dentists in NHS settings, because they had not been considered in the BDA inquiry in 1999, and in doing so, the opportunity was taken to retest BDA timings for face validity (see Table 1).

**Table 1:** **Panel inquiry timing results and BDA Heathrow Timings and total treatment demand 2011/12 England primary care**

	**BDA Heathrow Timings in minutes**		**Total number of clinical items**	
**Treatment**	**Timings for adults**	**Timings for children**	**Adults**	**Children**
Examinations	11.3	11.3	21,141,217	9,024,617
Scale and polish	15.1	15.1	885,253	12,281,036
Fluoride varnish	*4*	*5*	1,350,626	456,854
Fissure sealants	18.2	18.2	40,105	257,324
Endodontics	75.7	75.7	588,241	38,157
Tooth restorations	19.7	17.6	10,725,507	3,426,437
Extractions	17.6	17.5	2,936,211	902,049
Upper denture acrylic	76.4	76.4	600,702	3,743
Upper denture metal	79.1	79.1	54,924	363
Lower denture metal	79.1	79.1	28,291	61
Lower denture acrylic	76.4	76.4	362,817	723
Veneers	50.5	50.5	29,884	3,338
Inlays	50.5	50.5	188,238	10,825
Radiographs	*5*	*5*	10,561,103	949,485
Bridge units	63.7	63.7	184,553	5,638
Crowns	63.7	63.7	781,322	13,162
Antibiotic prescribing	*4*	*4*	532,806	68,894
Total			50,991,800	27,442,706

*Source*: BDA Heathrow Timings: panel inquiry treatments in italics.

**Figure 1:**
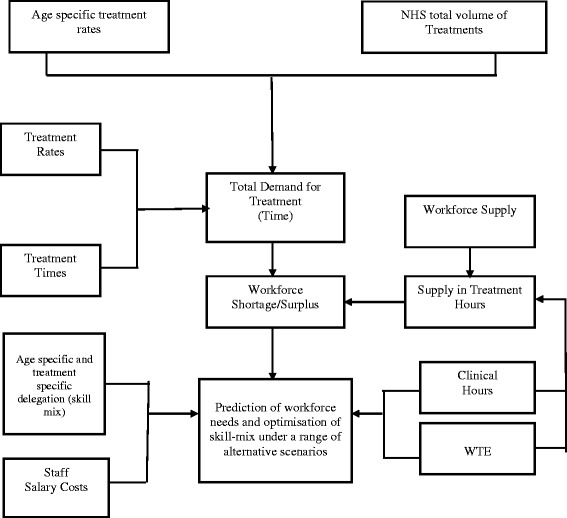
**DENTASSim model structure (demand, supply and optimization components).**

The scenario building and testing were based on changes to NHS practice and changing needs; this involved modelling NHS dental treatment data from the Business Service Authority (payment authority) for England in the year 2011/12. The data were presented for 17 listed treatments and were age-specific. The OR model was designed on an MS Excel spread sheet for ease of use and referred to as a *Den*tal *T*reatment *A*nd *S*kill mix *Sim*ulation model (DENTASSim). This is because the model incorporates elements of dental treatment and skill mix use and provides a basis for simulation. The DENTASSim model’s main output is age-specific clinical hours by provider dentist or dental therapist. The model and its components as they interact are shown in Figure 1. The components were influenced by literature [29,30], and delegation practices at the University of Portsmouth Dental Academy (UPDA) [31], as outlined above.

There were four NHS activity scenarios created whose outputs were clinical time, whole time equivalent (WTE), workforce numbers and salary costs on DENTASSim:

1. “No Skill Mix”, which modelled all care being undertaken by dentists and no delegation to dental therapists.
2. “Minimal Direct Access”, which involved simulating different levels of delegation of diagnostic tasks, with delegation ranging from 0–100% for dental examinations.
3. “More Prevention”, where evidence-based practice and skill mix were tested by increasing fluoride varnish treatments in children under the age of 12 from 13.1% of courses of care [32], which was the level of fluoride varnish in NHS activity for the year 2011–12, through to 100%, with care delegated at the rate during training (91%) to dental therapists and no direct access.
4. “Maximum Delegation”, where partial direct access is provided for patients, and contested care are undertaken by mid-level providers. Contested care – these are mainly medium-complexity treatments which can be undertaken by both dentists and dental therapists and are therefore contested when delegation is considered – they included tooth restorations, radiographs and tooth extractions on children. This scenario allocates 100% of examinations and prevention to dental therapists, 50% radiographs, 50% restorations and 50% children’s extractions to dental therapists. This is based on the rationale that it may be necessary for a dentist to retain patients for routine care, as well as technologically advanced care for complex patients, for example, medically compromised patients.

This was followed by a calculation of the likely NHS workforce numbers required for each scenario based on both the DENTASSim-simulated total WTE required to fulfil all demand in 2011/12 and established working patterns from a recent survey of dental professionals in England [30]. These working patterns were 0.4 WTE for dentists and 0.3 WTE for hygiene therapists (for clinical work only on the NHS). The figures were then compared with the number of dentists and dental therapists registered in England with the GDC. Following this calculation, a comparison of the salary cost for each scenario was undertaken by ascertaining the total salary for dentists and dental therapists at a rate outlined by salary rates from the National Career Service Advice [33].

## Results

Seventeen treatment groups represented the items of care undertaken by NHS primary care dental providers in 2011/12 as shown in Table 2, and simulation suggests that the total amount of clinical time required to undertake all these treatments based on model parameters was 19.3 million hours. The highest proportion of clinical hours was taken up by examinations at 5.7 million hours (29.4%), and the lowest was related to veneers at 27 962 h (0.1%). The table also shows that diagnostic procedures take the most clinical time. Medium-complexity treatments, which both dentists and dental therapists can undertake, were the second most time-consuming treatments. Prevention treatments commonly undertaken by DCPs constituted 18.4% of the clinical time. The model suggests that 77.6% of clinical time can be undertaken by dental therapists, while all dental care can be undertaken by dentists.

**Table 2:** **DENTASSim base model results – grouped treatments according to complexity**

**Treatment group**		**Clinical hours**	**Percentage of all clinical hours**	**Treatment group percentage of all clinical hours**
Diagnostic (can be undertaken by dentists or therapists and are contested treatments)	Examinations	5,681,232	29.4	34.4
	Radiographs	959,216	5.0	
Medium complexity (can be undertaken by dentists or therapists and are contested treatments)	Tooth restoration	4,567,989	23.7	24.8
	Paediatric tooth extraction	193,948	1	
	Paediatric endodontics	19,275	0.1	
Complex treatments (can be undertaken by dentists only)	Adult tooth extractions	930,954	4.8	22.2
	Crowns	843,477	4.4	
	Endodontic treatment	771,031	4.1	
	Upper denture acrylic	769,660	4.0	
	Lower denture acrylic	462,908	2.4	
	Bridge units	201,919	1.0	
	Inlays	167,545	0.9	
	Upper denture metal	72,887	0.4	
	Lower denture metal	37,377	0.2	
	Veneers applied	27,962	0.1	
Prevention (can be undertaken by dentists or therapists and are commonly delegated)	Scale and polish	3,313,516	17.2	18.4
	Fluoride varnish	143,009	0.7	
	Fissure sealants	90,220	0.5	
Other (can be undertaken by dentists only)	Antibiotic items prescribed	40,113	0.2	0.2

Complexity has been based on treatments within the skills of the dentists only.

Age-specific outputs, displayed in Table 3, suggest that the largest proportion of clinical time is undertaken on 45–54 year olds (16.2%), and the least is for those aged between 0 and 2 years (0.6%).

**Table 3:** **DENTASSim base model results – total clinical hours to meet demand by age group**

**Age groups**	**Clinical hours**	**Proportion of total hours (%)**
**0–2 years**	125,281.4	0.6
**3–5 years**	459,936.4	2.4
**6–12 years**	171,342.3	8.9
**13–17 years**	129,443.7	6.7
**18–24 years**	1,319,555	6.8
**25–34 years**	2,355,367	12.2
**35–44 years**	2,881,285	14.9
**45–54 years**	3,120,443	16.2
**55–64 years**	2,738,878	14.2
**65–74 years**	2,015,668	10.4
**75+ years**	1,269,964	6.6
**Total hours**	19,294,238	

Scenario 1: “No Skill Mix” outputs suggested that all base model clinical times would be undertaken by dentists only, and this would amount to a need for 12 685 WTE dentist, working to undertake 19.29 million clinical hours. The total average salary cost would be £750.1 million.

Scenario 2: “Minimal Direct Access” scenario showed the impact of sharing examinations between dentist and dental therapist/DCP in 10 different proportions between 0% and 100%. The findings are presented in Table 4. According to this scenario, once 70% of examinations are delegated to DCPs, and other treatments are delegated at a rate similar to the primary care training site in the South of England, a 1:1 WTE ratio of dentist to dental therapist is required, with equal clinical hours performed by both groups.

**Table 4:** **DENTASSim scenario 2 “Minimal Direct Access” clinical hours by % of examinations undertaken by dental therapists**

**% of examinations undertaken by dental therapists**	**Proportion of clinical time by dentists for all clinical hours (%)**	**Dentists total clinical hours**	**Proportion of clinical time by dental therapists for all clinical hours (%)**	**Dental therapists total clinical hours**
0	70.5	13,610,437	**29.5**	**5,683,801**
10	67.6	13,042,313	**32.4**	**6,251,925**
20	64.7	12,474,190	**35.3**	**6,820,048**
30	61.7	11,906,067	**38.3**	**7,388,171**
40	58.8	11,337,944	**41.2**	**7,956,294**
50	55.8	10,769,821	**44.2**	**8,524,418**
60	52.9	10,201,697	**47.1**	**9,092,541**
*70*	*49.9*	*9,633,574*	***50.1***	***9,660,664***
80	47.0	9,065,451	**53.0**	**10,228,787**
90	44.0	8,497,328	**56.0**	**10,796,910**
100	41.1	7,929,205	**58.9**	**11,365,034**

Age-specific clinical time in hours for dental therapists are in bold, and the regular font represents dentists’ clinical hours.

**Figure 2:**
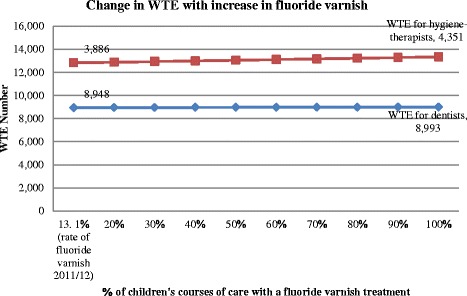
**DENTASSim scenario 3 “More Prevention” overall change in WTE.** Note: *orange* represents age-specific clinical time in hours for dental therapists while *blue* represents dentists’ clinical hours.

Scenario 3: “More Prevention” involved improving evidence-based practice by increasing fluoride varnish. The results displayed in Figure 2 indicate that if 100% of fluoride varnishing was performed on all children’s course of care, there would need to be an increase of 465 WTE of dental therapists or other DCPs who can perform fluoride varnish compared to 45 WTE dentists.

Scenario 4: “Maximum Delegation”, where a high proportion of contested care and all examinations are delegated, resulted in findings that suggest that 4801 WTE dentists and 8199 dental therapists would be required to meet the total demand for care as displayed in Table 5.

**Table 5:** **All scenarios cost minimization and workforce numbers outputs**

	**Dental professional**	**DENTASSim clinical time in hours**	**DENTASSim WTE**	**Estimated NHS number of personnel required to meet total demand**	**Ratio of NHS personnel dentist to hygiene therapist**	**Minimum salary cost**	**Average salary cost**	**Maximum salary cost**	**% salary cost saving between no skill mix and other scenarios**
Scenario 1: “No Skill Mix” (dentists only)	Dentists only	19,294,238	12,685	24,736		**£478,410,847**	**£750,889,634**	**£1,023,368, 422**	0%
Scenario 2: “Minimal Direct Access” 70% exams delegated +UPDA delegation rate	Dentists	9,633,574	6,334	12,351	1:1.3	£238,869,572	£374,917,681	£510 965,790	
	Dental therapists	9,660,664	6,606	16,513		£140,038,342	£185,616,859	£231,195,376	
Total				**28,864**		***£378,907,914***	***£560,534,540***	***£742,161, 167***	38%
Scenario 3: “More Prevention” 50% of children receive fluoride varnish +UPDA delegation rate	Dentists	13,623,505	8,967	17,466	1.8:1	£338,185,478	£530,798,939	£723,412,400	
	Dental therapists	5,815,934	4,084	9,941		£86,572,892	£114,749,918	£142,926,945	18%
Total				**27,407**		***£424,758,370***	***£645,548,858***	***£866,339, 345***	
Scenario 4: “Maximum Delegation” (delegation of 100%: examinations prevention, 50% of radiographs, tooth restoration and children’s extractions = 62.8% of total clinical time)	Dentists	7,290,502	**4,801**	9,346	1:2	£181,073,278	£284,203,521	£387,333,764	
	Dental therapists	11,913,515	**8,199**	20,365		£173,826,662	£230,402,321	£286,977,980	52%
Total				**29,711**		***£354,899,939***	***£514,605,842***	***£674,311, 744***	

Salary source is the National Careers Service: https://nationalcareersservice.direct.gov.uk/Pages/Home.aspx [33].

Number of personnel is calculated based on working hours described by Robinson et al. [30], that is, WTE for clinical time (dental therapist 0.3 WTE; dentist 0.4 WTE).

Table 5 compares all scenarios against scenario 1 “No Skill Mix” and also includes salary cost evaluation. The model findings here suggest that there would be salary costs saved through the use of skill mix. First, direct access at the rate of 70%, which was part of scenario 2 “Minimal Direct Access” simulations, which simulated a need for 6334 WTE dentists and 6606 WTE dental therapists, also displayed in Table 5, when adapted to established NHS working patterns, would equate to 12 351 dentists to be involved in NHS care along with 16 513 dental therapists. And this number of NHS personnel would lead to 38% salary cost saving compared with a “No Skill Mix” scenario. These workforce numbers further suggest that 40% of registered dentists and eight times the number of dental therapists currently registered in England would be needed to be involved in provision of care.

Using the same parameters to establish salary cost, the findings displayed in Table 5 also suggest that increasing fluoride varnish from 13% to 50% in the “More Prevention” scenario and maintaining the training facility model of skill mix would require 4.7 times the number of dental therapists and 57% of registered dentists; this would represent a 1% salary cost saving compared with a “No Skill Mix” scenario which maintained a 13.1% rate of fluoride varnish treatments.

While in the case of scenario 4 “Maximum Delegation”, with all care within dental therapists’ jurisdiction delegated to them, except for restorations, children’s tooth extractions and radiographs (50%), which represent 62.8% of the total clinical time, the findings suggest that only 30% of registered dentists would be required and 10 times the number of dental therapists registered. This scenario could achieve a 52% salary cost saving compared with “No Skill Mix”.

## Discussion

The findings from this study advance knowledge by quantifying possible contributions of dental therapists or equivalently trained mid-level dental providers in the provision of evidence-based prevention in primary dental care in England, based on current activity and salary costs. This work advances the research in the field by including the whole population age spectrum, in comparison to past research that has concentrated on older people’s oral health needs and demand [23]. This work also differs from on-going studies which are focussing on technical efficiency and specifically the production of the maximum amount of output from a given amount of input [16], in that it not only explores both production of outputs (dental care) using a skill-mix-oriented workforce but undertakes a further investigative view towards evidence-based prevention using skill mix. In recognition of the importance of the business model in dental practice [13,34], this research also includes an economic evaluation of skill mix, using a salary cost minimization evaluation, thereby covering some of the main aspects implicated for their influence on the use of skill mix.

The results suggest that only around 23% of clinical time was spent on “dentist only” tasks within the NHS nationally. This provided evidence of the limited exclusive jurisdictional claim between dentists and dental therapists. It demonstrates that the majority of care undertaken in NHS primary dental care in England may potentially be performed by dental therapists. These findings compare well with estimates made by Harris and Burnside in 2004 [35] and the Nuffield Foundation in 1993 [36] who suggested that approximately 80% of tasks in the NHS can be undertaken by dental therapists.

This research also identifies the treatments and patient groups that accounted for the most clinical time by ascertaining working time in relation to volume of tasks. Such studies have been shown to be useful in establishing the most time-consuming tasks or patient groups in public dental services [37-39]. The results of the current study compare with previous studies, by demonstrating that “diagnostic tasks” consume most clinical time. In this study, these represented 34% of clinical time, of which 86% of the diagnostic time was spent undertaking examinations and care planning; similar findings were suggested by Evans et al. in 2007 [40] in NHS general dental practice in Wales. The time taken for diagnostic tasks presents as very relevant to the skill mix debate, as regulation changes have evolved slowly when considering DCPs as the first point of contact, ergo as the diagnostician. Now that “Direct Access” to DCPs is allowed in the UK, the potential to save clinical time through delegation of these tasks is large, as suggested by this model, with the important provision that they work at the same speed as dentists.

In relation to the implementation of sharing of diagnostic tasks in NHS state-funded dental care in England, these findings highlight a challenge to rectify the mismatch between regulations from different bodies which limits the use of DCPs in state-funded care in England. This is in reference to the NHS policy that does not yet allow DCPs to hold performer numbers and claim for NHS care, which is similar to the challenge faced by clinical dental technicians [41]. In addition, there is evidence generated regularly on the reliability of DCPs’ diagnostic skills [12], further emphasizing the need to facilitate this process of task sharing.

Other results from this study suggested that patients aged between 45 and 54 years were the most time-consuming age group (16.2% of all clinical time). Children’s clinical times were all lower than adult times and only compared with over 75 year olds who required only 6% of all the clinical time. Several studies have indicated variation in care in different age groups [42-44]. The findings in this study take account of shifting patterns of disease and population oral health needs [8,9], the need in England and the strategic review of dental services by Steele in 2009 [45], which together suggest a rise in complex care needs amongst middle-aged groups. A shift in the oral health profile in populations with an increased need for complex treatments which take longer in these middle-aged groups has now been seen across Europe [8]. The significance of this finding lies in the understanding of where demand is likely to be found and how time may be freed up and shared for optimal efficiency.

Evidence-based practice and skill mix were tested using scenario 3 “More Prevention”, which increased fluoride varnish for children, which is a recommended preventive measure advocated in delivering better oral health: an evidence-based toolkit for prevention [7]. In the year 2011/12, the delivery of fluoride varnish described in the data used in the model constituted 13.1% of completed courses of care undertaken on children [32]. The simulation findings in this current study suggested that with an increase from 13.1% to 100% fluoride varnish courses of care undertaken on children and 91% delegation rate of fluoride varnish to dental therapists, only an extra 465 WTE dental therapists and 45 WTE dentists would be required. This initiative can be taken forward without applying direct access arrangements, that is, delegating examination. It is important to note that this translated to only a 1% increase in salary costs. This is the first analysis on the impact of increasing fluoride varnish based on evidence-based recommendations and with the use of skill mix. This relates back to the points highlighted in the literature regarding how skill mix can contribute to effective health promotion strategies [46,47]. This is worth considering as it has been highlighted that there is room for long-term improvements in oral health as a result of prevention, which would lead to longer term cost savings [48]. The methods used in this study indicate that other preventive procedures such as fissure sealants and how skill mix can impact on the provision of these treatments can also be reliably undertaken.

The final model scenario “Maximum Delegation” was an underestimate of what dental therapists could perform but on the higher end of skill mix use. It applied 100% delegation of some treatments to DCPs: examinations and prevention, and 50% delegation of contested care: radiographs and tooth restoration, and based on these rates of delegation and the DENTASSim simulation, this would account for 62.8% of all clinical time. With even this underestimation of delegation, the model suggested a potential 52% salary cost saving with this “Maximum Delegation” model compared with “No Skill Mix”. There are implications for the workforce with these findings, as according to the WTE generated by the DENTASSim based on the working patterns described by Robinson et al. in 2011 [30], there would be a need for 20 365 dental therapists. Presently, there are 2128 dental therapists and 5462 hygienists, and some of the hygienists are dually trained and registered under both professional groups [49]. It is therefore realistic to estimate that on the lower side, if hygienists undertook some of the tasks, there would be a need for at least four times the number of therapists (9941), and with “Maximum Delegation”, 10 times the present number of dental therapists (20 365) would be required to meet demand. However, application of fluoride varnish does not require the skills of a dental hygienist or therapists, as dental nurses may gain additional skills in oral health education and fluoride varnish application and thus undertake these preventative tasks which constituted 18.4% of clinical time. This would potentially lead to even higher salary cost savings. It must, however, be noted that these figures are based on maintaining the demand and working rates described in DENTASSim model, of which either could increase or decrease. And as professional groups expand their scope of practice, they are likely to demand higher salaries, as a likely outcome is that the cost differential by task delegation decreases over time [50]. Further research is required on appropriate delegation patterns to inform workforce decision making.

### Implication for education and practice

The implications of this scenario modelling can translate to education and training and professional growth for DCPs. The findings point to the benefit of training more DCPs, especially if more prevention is to be undertaken and if time is to be made available for future complex care. However, factors such as DCPs’ part-time work arrangements after family commitments [51] may have an impact on the actual amount of contributions they would make. Furthermore, there is an indication that training places for dentists need to reduce as supported by other research and NHS plans [25,52]. The findings provide a representation of how DCPs’ skills could be maximized and can be used to encourage mid-level providers to see that they can play a bigger role in the delivery of primary dental care and a change in the dental workforce, subject to appropriate funding models for training and delivery of care.

### Implications for research

It is clear that there is a need for more evidence nationally to accurately ascertain how much time DCPs are working, at what rates and rationale for delegation (upwards and downwards) as new models of care emerge. This can be done by service evaluation studies, supported by patient management systems. The findings from this skill mix analysis provide a good foundation and insight on how this can be achieved [53].

### Implications for policy

Some of the findings arising from the research translate back to the roles of DCPs and their personal development as a professional group. As Sanglard-Oliveira et al. in 2012 [54] argued, for a group to be considered a professional entity, it needs to be able to control its own work and this relates to autonomy which is legitimized by society and regulated [55]. For DCPs in England, the public have been accepting of their roles, as individuals have cited “trust” of the dental professional as more important rather than whether he/she is a DCP or a dentist [56]. With regulations supporting their expanded roles, the policies in their work environment, and most notably the funding mechanisms [13], are the only area that needs development in order to grow the roles of DCPs.

Wake in 2014 [57] rightly suggests that the direct access plans only facilitate better working within general practice and do not signify opportunities to setup rival practices against dentists, as several other elements of patient care are still regulated under dental leadership, such as the prescribing of drugs. It is, however, a challenge for general practitioners, many of whom are practice leads and “providers” holding NHS contracts and performer numbers, to envisage the benefit of including DCPs in care. Bullock and Firmstone in 2011 [58] suggest that perhaps one of the bigger challenges for skill mix development in primary care is the large number of GDPs who are likely to “close ranks” and thereby challenge the process of role development. The question of “turf wars” is a pertinent one when attempting to rearrange traditional boundaries of professionals [59]; for any substantial debate, ethical and economic arguments can be presented, based on research such as this. Wide professional and economic debate has ensued with regard to the use of dental therapists [53,60-62] and evidence from Finland and the USA suggesting minimal economic benefit for DCPs [62,63]. It is worth mentioning that cost in relation to increased use of skill mix is context driven and complex, constituting a variety of proponents such as estate cost and materials cost [15]. In this study, one aspect of cost is considered – salary cost – and its limitation is recognized. However, it focuses on a critical aspect of cost and provides a useful base of understanding for practice leads who have been shown to demonstrate variation and an uncertainty in remuneration arrangements that improve the use of skill mix [13,35].

In terms of ethics, for those who subscribe to the theory of equity in health, such as egalitarian liberals or communitarians, the potential for quality care being equally divided through prevention and synergy in the practice of DCPs and dentists has been demonstrated. Equally, this does demonstrate a potential cost saving, although this does not encompass all aspects of cost as highlighted, but the opportunity to redistribute resources based on savings from one domain such as salaries is encouraging, provided they are reinvested to patient and public benefit.

### Strengths and limitations

The use of patient management and national payment data to ascertain who provides what care demonstrates a non-invasive and reliable way to undertake this work. However, there were some limitations to the operational research exercise. First, the model components on delegation rates are based on a single site. That said, single-site studies are valid, as they provide an opportunity to undertake in-depth investigations on the phenomenon as it occurs naturally [64]. Second, that it was an educational establishment; however, the fact that the facility in question used a live NHS contract constitutes a legitimate example of how skill mix can be practised. Although generalizability of findings is a common limitation of single-site studies, to mitigate these, robust protocols and validating criteria prescribed by experts in the field of research were adopted [64].

The operational research model, DENTASSim, was able to provide definitive results on distinct scenarios and age- and treatment-related outputs; however, it would have been useful to have included other variables in the model that describe other social parameters that do impact on need for care. Unfortunately, there were no data on parameters such as deprivation (or postcode for calculation of deprivation) available as part of the national dental data. This is an area for further research working in collaboration with the NHS.

The next step for dental therapists and other DCPs is appropriate education and training to fulfil the range of tasks that the prevailing needs of the population are demanding. More work is required to estimate how the change in needs is progressing through the years and how education numbers would need to change. Presently, there is already a drive to reduce training for dentists by 10% and increase dental therapists’ training spaces [52]. While there is good rationale for developing the skill mix of the dental team, on-going research to guide this process is important.

## Conclusion

The operational research findings suggest that if demography, task sharing and working time are included as predictors of demand on dental services, the clinical time and workforce requirements can be identified to the detail of patient group and treatment. When these aspects of demand are altered to reflect reorganization of working relationships based on regulation changes that improve autonomy and jurisdiction (direct access) of DCPs such as dental therapists, learning can be gained on the distribution of care activities. It suggests that there is potential to expand the roles of other DCPs such as dental nurses with extended duties as part of future workforce developments.

## Abbreviations

DCPs: Dental care professionals
UPDA: University of Portsmouth Dental Academy
DENTASSim: *Den*tal *T*reatment *A*nd *S*kill mix *Sim*ulation model
NHS: National Health Service
WTE: Whole time equivalent
